# The Syracuse Hahnemannian Club

**Published:** 1888-04

**Authors:** 


### THE SYRACUSE HAHNEMANNIAN CLUB.

February 24th, 1888, a meeting was held at the office of Dr. William A. Hawley, Syracuse, for the purpose of organizing this Club, its object to be the study of the *Organon.*

The following request for the meeting was sent out:

"We, the undersigned, desiring to acquire a more thorough knowledge of pure Homœopathy as taught by Hahnemann, of the *Organon,* materia medica, and symptomatology, do hereby agree to assist in organizing a local society to be known as the 'Syracuse Hahnemannian Club,' meetings to be held weekly at such times and places as may be agreed upon at the first meeting. The meeting for organization is to be held at No. 52 Warren Street, on Friday evening, February 24th, at eight o'clock."

The call was signed by Drs. J. W. Sheldon, E. J. Robinson, E. O. Kinne, Frederick Hooker, G. N. Macomber, R. S. True, S. Seward, J. W. Candee, E. N. Flint, A. B. Kinne, H. D. Emens, William A. Hawley, F. B. Putnam, C. Shumacher, John Nottingham, A. J. Brewster.

The meeting was called to order with Dr. R. S. True in the chair. A Committee on Organization, consisting of Drs. S. Seward, A. B. Kinne, William A. Hawley, J. W. Candee, and E. J. Robinson, was appointed to prepare a programme for the future guidance of the Club, to be presented at the next meeting. Dr. S. Seward was then elected President, and Dr. Frederick Hooker Secretary. It was decided to begin the study of the *Organon* at the next meeting, and Dr. True was invited to give the clinical relations of Sepia as found in his own experience.

March 2d the regular meeting of the Syracuse Hahnemannian Club was held at Dr. R. S. True's office in the Crouse building.

The Committee on Organization, appointed at the last meeting, presented the following preamble and resolutions:

"WHEREAS, The Syracuse Hahnemannian Club is organized for the sole purpose of studying the fundamental principles and philosophy as unfolded in Hahnemann's *Organon* and other writings, and its materia medica; therefore,

"*Resolved*, That its officers shall be a President and Secretary, who shall be elected semi-annually (the last Friday in February and the last Friday in August), and their duties shall be those common to those offices in deliberative bodies; further

"*Resolved*, In order that all may have equal opportunity to express their ideas concerning any point under discussion, no one shall occupy more than five minutes on any one speaking, nor shall he speak more than twice on the same question, except by vote of the Club, and that vote shall fix the limit of time he may further occupy; further

"*Resolved*, That any graduate of medicine, of good standing, desirous of pursuing these proposed studies, may become a member of this Club."

The report was unanimously adopted. After some preliminary discussion the Club proceeded to the consideration of Section 1 of the *Organon*. After a spirited and profitable discussion, Dr. R. S. True read an able paper, entitled "In the Light of Pure Homœopathy, What I Know About Sepia." The paper was well received, and the discussion that followed was of interest to all present. The Club then adjourned to meet at Dr. E. J. Robinson's office, No. 82 South Salina Street, on Friday evening, March 9th, at eight o'clock.

A fuller report of the proceedings will be given in next issue.

